# Amino-Fe_3_O_4_ Microspheres Directed Synthesis of a Series of Polyaniline Hierarchical Nanostructures with Different Wettability

**DOI:** 10.1038/srep33313

**Published:** 2016-09-16

**Authors:** Yong Ma, Yanhui Chen, Chunping Hou, Hao Zhang, Mingtao Qiao, Hepeng Zhang, Qiuyu Zhang

**Affiliations:** 1 Key Laboratory of Applied Physics and Chemistry in Space of Ministry of Education, School of Science, Northwestern Polytechnical University, Xi’an 710072, P. R. China

## Abstract

We demonstrated polyaniline (PANI) dimensional transformation by adding trace amino-Fe_3_O_4_ microspheres to aniline polymerization. Different PANI nanostructures (i.e., flowers, tentacles, and nanofibers) could be produced by controlling the nucleation position and number on the surface of Fe_3_O_4_ microspheres, where hydrogen bonding were spontaneously formed between amino groups of Fe_3_O_4_ microspheres and aniline molecules. By additionally introducing an external magnetic field, PANI towers were obtained. These PANI nanostructures displayed distinctly different surface wettability in the range from hydrophobicity to hydrophilicity, which was ascribed to the synergistic effect of their dimension, hierarchy, and size. Therefore, the dimension and property of PANI nanostructures can be largely rationalized and predicted by adjusting the PANI nucleation and growth. Using PANI as a model system, the strategies presented here provide insight into the general scheme of dimension and structure control for other conducting polymers.

Nanostructured conducting polymers combing the electrical properties of metals with the ease of processability have attracted considerable attention since the 1970s^1^,^2^. Within the large family of conducting polymers, polyaniline (PANI) is one of the remarkable polymers and finds a broad band of applications in chemical sensors, electrochromic devices, energy storage, etc., owing to its intriguing reversible acid/base doping/dedoping chemistry and tunable conductivity^3^,^4^,^5^. Many studies have lately focused on the preparation of PANI nanostructures due to the magnified or enhanced properties compared to their bulk counterpart^6^. For example, PANI nanofibers in sensors gave significantly better performance in both sensitivity and time response compared to conventional PANI films^7^,^8^. PANI nanowires combining with graphene oxide exhibited immense potential for preparing supercapacitors with high capacitance energy storage^9^,^10^. PANI/carbon nanotubes hybrid film as flexible energy devices improved energy density without deteriorating their high power capability^11^. It is noteworthy that in above cases PANI dimension is a key parameter to determine its performance and application. Therefore, the delicate regulation and control of PANI dimension is highly desirable.

There are some pioneering works in which PANI can vary its dimension with the aid of additives. Seeding the aniline polymerization with trace biological, inorganic, or organic nanofibers changed the PANI morphologies from particulates to almost exclusively nanofibers^12^. Right- and left-handed helical PANI nanofibers were acquired using _D_- and _L_-CSA as the dopant, respectively^13^. Hexagonal superlattice and nanospheres of chiral PANI were fabricated by separately mimicking *β*-sheet proteins^14^ and bovine hemoglobin or bovine serum albumin^15^. PANI leaf-like structures were obtained after adding F127 to the polymerization system, while only large particulates were prepared in the absence of F127^16^. In a separate study, PANI nanorings and flat hollow capsules were obtained by applying VOPO_4_·H_2_O nanoplates^17^. These successful realizations of PANI dimension control are a result of that aniline or PANI molecules are subjected to the guidance, mimicking, or micelle effects derived from the various additives, of which the functional groups usually play a decisive role in directing PANI molecules orchestration. But as of yet, sparse reports have concentrated on the effect of quantity of functional groups of additives on the PANI dimension and the resulting properties.

In this study, PANI flowers, tentacles, and nanofibers were separately synthesized in a mild reaction conditions (in 0.010 M HCl solution at room temperature), by means of adding minute Fe_3_O_4_ microspheres modified with different quantity of amino groups. The growth process of these nanostructures was clarified, for which the significance of amino groups was also illuminated. In addition, PANI tower-like morphology was formed by additionally introducing an external magnetic field, and its formation mechanism was also discussed. The films of these resulting PANI nanostructures exhibited diverse surface wettability, which are quite promising to be corrosion resistant coatings, electrostatic shielding materials, and chemical sensors.

## Results and Discussion

**Figure 1:**
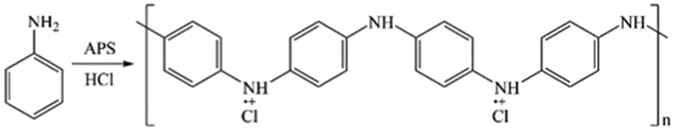
Reaction for the chemical oxidative polymerization of aniline with APS acting as the oxidant in HCl solution.

**Figure 2:**
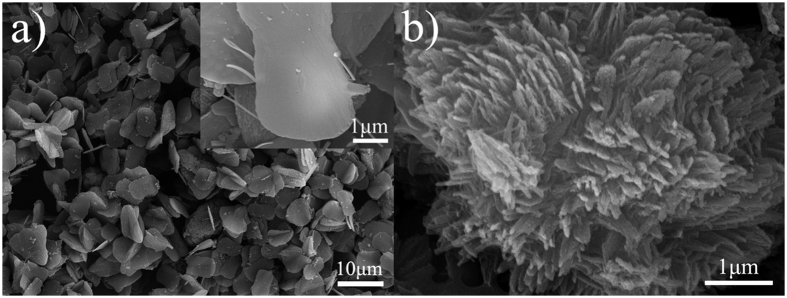
(**a**) SEM image of PANI bulks (plate- and grass-like structures) obtained by chemical oxidative polymerization of aniline, inset (**a**) and (**b**) images of magnified plate- and grass-like structures, respectively.

The synthesis process of PANI is illustrated in Fig. 1, in which APS serves as oxidant and HCl solution works as the reaction medium. Figure 2(a) shows SEM image of PANI bulks synthesized by chemical oxidative polymerization in 0.010 M HCl solution. It is evidently viewed that the bulks consist of a large number of two dimensional (2D) plate-like strucftures similar to shoe soles with thickness of about 270~700 nm and lateral diameter of 3.0~4.2 μm × 4.7~7.1 μm, and a handful of three dimensional (3D) grass-like structures with size of several microns. At a closer observation, plate-like structure possesses a smooth surface (inset image in Fig. 2(a)), and grass-like structure is made up of unordered nanofibers with 36~75 nm in diameter and several microns in length (Fig. 2(b)). As previously reported in the literature, PANI from plate-like structures to grass-like superstructures were tailored in an appropriate molar ratio (0.1:1–0.8:1) of oxidant to monomer^18^.The appearance of these PANI plates is ascribed to the quick precipitation of freshly formed PANI with insufficient doping in a low acid environment (pH = 2)^19^. For the grass-like structures, their formation is attributed to the secondary growth of PANI. In the reaction system, although almost PANI plates can be easily precipitate from solution, a small number of plates act as the platform and place for PANI nucleation and growth. Moreover, due to the competitive growth of nucleation and in an attempt to reduce the high interfacial energy between the freshly formed PANI agglomerates and water molecules, the secondary growth favors fibrous-like morphology.

**Figure 3:**
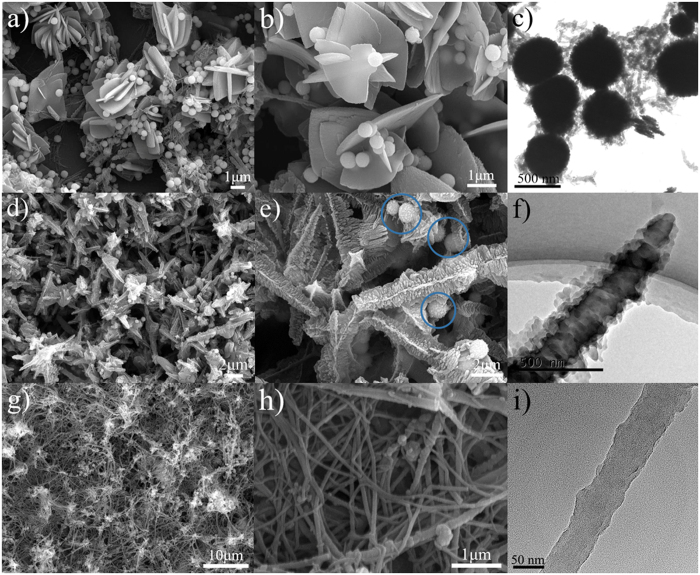
(**a**,**b**) SEM images of PANI flowers obtained by adding Fe_3_O_4_ microspheres, (**c**) TEM image of Fe_3_O_4_ microspheres and short PANI nanofibers; (**d**,**e**) SEM and (**f**) TEM images of PANI tentacles obtained by adding (**I**) amino-Fe_3_O_4_ microspheres; (**g**,**h**) SEM and (i) TEM images of PANI nanofibers obtained by adding (II) amino-Fe_3_O_4_ microspheres. The N atomic percent of three kinds of microspheres is 0, 2.81, and 4.34%, respectively (supporting information Figure S1–S3).

Interestingly, when adding trace Fe_3_O_4_ microspheres prepared by the solvothermal method^20^ into the foregoing PANI polymerization, 3D flower-like PANI composed of sheets having thickness of 145~360 nm and lateral diameter of 0.8~1.2 μm × 1.2~2.4 μm concomitant with nanofibers are produced, as shown in SEM image of Fig. 3(a). From the magnified SEM image of Fig. 3(b), independent smooth Fe_3_O_4_ microspheres with about 400 nm in diameter are clearly seen to decorate the sheets of PANI flowers. In TEM image of Fig. 3(c), it is distinctly observed that no PANI coating shell forms on the periphery of Fe_3_O_4_ microspheres and only some scattered short PANI nanofibers surround them, which convincingly verify a fact that PANI nucleation process occurs in the solution rather than in the surface of Fe_3_O_4_ microspheres. The formation of these flowers is thought to be a consequence of space-occupying effect of Fe_3_O_4_ microspheres. Although the hydrophilic Fe_3_O_4_ microspheres are well dispersed in the reaction system, their slow settlement on one hand occupies PANI growth space, on the other hand accelerates PANI precipitation process. Hence, the newly formed PANI has to grow, propagate, and terminate in a compact way. With increasing polymerization time, these gathered PANI assembles, coagulates, and fuses with each other, resulting in the formation of PANI flowers. During the polymerization process, quite a few long-chain oligomers may be absorbed to the surface of Fe_3_O_4_ microspheres which still disperse well in the solution. The continuous growth of these oligomers in a non-occupied environment beneficially presents their intrinsically linear nature^21^,^22^,^23^. Accordingly, PANI nanofibers adhering on the surface of Fe_3_O_4_ microspheres are simultaneously yielded.

In another Fe_3_O_4_ microspheres system ((I) amino-Fe_3_O_4_ microspheres), the resulting PANI in 2D tentacle-like morphology is unexpectedly observed for the first time. From SEM image of Fig. 3(d), one can find that PANI tentacles have 370~765 nm in width and 2.9~4.5 μm in length. A white straight line can be viewed in the central axis of every tentacles. Magnified SEM image (Fig. 3(e)) highlights the detailed composition of the tentacles in which many protuberances with the height of tens of nanometers occupy the central axis and a myriad of flakes are vertically arranged along their central axis. Moreover, Fe_3_O_4_ microspheres having coarse surface are clearly shown (marked in circles), which means that there are PANI formed on their surface. This phenomenon is sharply different from the above situation where pristine Fe_3_O_4_ microspheres are employed. In TEM image of Fig. 3(f), the tentacle structure is solid and has countless prominent flakes arrayed laterally on its surface, in accordance with the SEM images. Note that its top is thinner than the bottom, indicating there is a cutting edge appearing during the tentacle growth.

When adding (II) amino-Fe_3_O_4_ microspheres, amazingly, the obtained PANI is in shape of one-dimensional (1D) nanofibers having length above 10 μm and diameter in the range of 35~141 nm, as displayed in SEM image of Fig. 3(g,h). In TEM image of Fig. 3(i), the solid nanofiber has a relatively smooth surface and only a few protuberances with low curvature on its periphery. The PANI coating shell can be clearly observed on the surface of (II) amino-Fe_3_O_4_ microspheres (supporting information Fig. S4), which differs from the case of using pure Fe_3_O_4_ microspheres where these microspheres are surrounded with short PANI nanofibers. The formation of PANI coating shell demonstrates there is a strong interaction between (II) amino-Fe_3_O_4_ microspheres and aniline molecules. We think that PANI morphology dominated by nanofibers is attributed to its intrinsically linear nature^21^,^22^,^23^.

**Figure 4:**
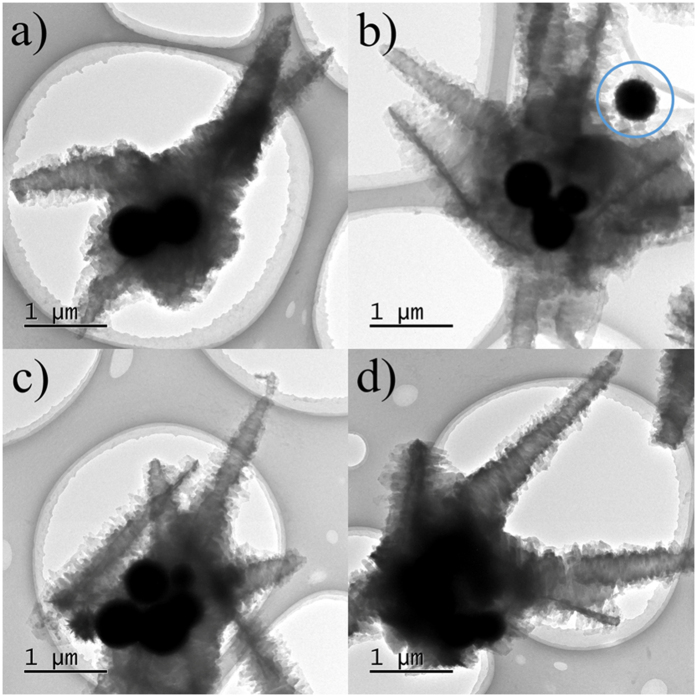
TEM images of PANI tentacles with different numbers of Fe3O4 microspheres in their interior: (**a**) two; (**b**) four; (**c**) five; (**d**) a pile of Fe_3_O_4_ microspheres.

To elucidate the mechanistic rationale of the special tentacle structures, different tentacle agglomerations are displayed in TEM image of Fig. 4. Two, four, five, and a pile of Fe_3_O_4_ microspheres can be distinctly observed in the junction and interior of these corresponding agglomerations. This phenomenon strongly demonstrates that Fe_3_O_4_ microspheres not only provide the position for PANI nucleation, but also support the platform for PANI growth. In particular, a single Fe_3_O_4_ microsphere coated with PANI coronals (marked in circle, Fig. 4(b)) is able to deduce the initial PANI growth process. During the polymerization, aniline molecules are adsorbed on the vicinity of (I) amino-Fe_3_O_4_ microspheres due to the effect of hydrogen bonding spontaneously formed between amino groups of Fe_3_O_4_ microspheres and aniline molecules^24^. This adsorption results in a great increase of the local concentration of aniline molecules near (I) amino-Fe_3_O_4_ microspheres. After APS oxidant is added, Fe_3_O_4_-PANI coronal composites gradually appear. The adhesion phenomenon of multi-Fe_3_O_4_ microspheres happens concurrently as a consequence of their Brownian movement. Since the subsequent polymerization takes place preferentially and continuously in the close proximity of already existing PANI owing to the low nucleation energy^25^, the newly formed PANI grows on the surface of the coronals instead of in the solution. Consequently, PANI tentacle agglomerations with multi-Fe_3_O_4_ microspheres in their inside are fabricated.

**Figure 5:**
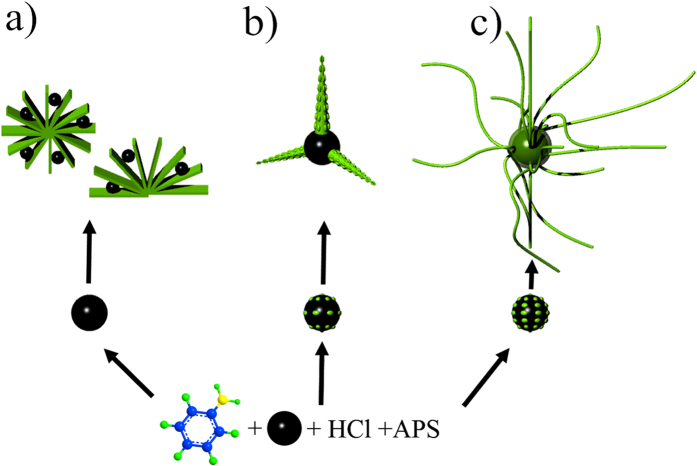
Schematic illustration of growth process of PANI (**a**) flowers, (**b**) tentacles, and (**c**) nanofibers.

We have already manifested that employing Fe_3_O_4_ microspheres with different quantity of amino groups obviously determine the dimension of the resulting PANI structures. In Fig. 5(a), on account of the lack of amino groups, pristine Fe_3_O_4_ microspheres just play a role in occupying space and have no direct effect on the PANI growth. Once in the presence of limited amino groups ((I) amino-Fe_3_O_4_ microspheres, Fig. 5(b)), only a small number of hydrogen bonding form, which make PANI selectively polymerize and deposit on the surface of Fe_3_O_4_ microspheres. The illustration of possible hydrogen bonding effect between amino-Fe_3_O_4_ microspheres and aniline molecules is shown in supporting information Fig. S5. Sequentially, PANI coronals form and distribute unevenly on the surface of Fe_3_O_4_ microspheres. These coronals continue to grow into PANI backbones under the effect of cutting edge. At the same time, new nucleation sites emerge on the backbones and PANI secondarily grow circularly perpendicular to these backbones, leading to the formation of PANI tentacles. As the polymerization proceeds with abundant amino groups ((II) amino-Fe_3_O_4_ microspheres, Fig. 5(c)), the formation of a large number of hydrogen bonding yields plenty of nucleation sites on the periphery of Fe_3_O_4_ microspheres. So many nucleation sites competing for growth consume almost all aniline monomers in a short period of time, which is beneficial to PANI linear growth^21^. Meanwhile, such behavior prevents PANI from secondary growth. As a result, PANI nanofibrous morphology are prepared.

**Figure 6:**
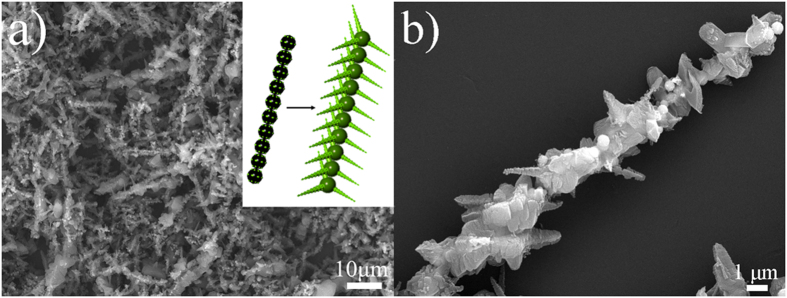
(**a**,**b**) SEM image of PANI towers obtained by using (I) amino-Fe_3_O_4_ microspheres under an external magnetic field, inset image in (**a**) of schematic illustration of PANI tower growth process.

Fe_3_O_4_ microspheres often impart a tolerance to head-to-tail arrangement along the magnetic force lines under a magnetic field, because they undergo a process of magnetic field induced self-assembly^26^,^27^. So it is promising to enrich the PANI nanostructures when utilizing (I) amino-Fe_3_O_4_ microspheres under an external magnetic field in the polymerization. As we expected, a fantastic PANI tower-like structure appears with length up to tens of microns, as shown in SEM image of Fig. 6(a). In SEM image of Fig. 6(b), a magnified image clearly exhibits there are PANI tentacles on the surface of PANI towers. More SEM images can be seen in the supporting information Fig. S6. Obviously, the appearance of this structure is dependent on the use of (I) amino-Fe_3_O_4_ microspheres associated with their self-assembly behavior under the magnetic field. Inset image schematically displays the evolution process of PANI tower. Upon the magnetic field, Fe_3_O_4_ microspheres easily assemble into chains in a head-to-tail way. There are PANI coronals gradually generated on the surface of Fe_3_O_4_ microspheres due to the formation of sparse hydrogen bonding as described above. Then, these coronals further grow into tentacles, which at the meantime immobilize the Fe_3_O_4_ microsphere chains. As time goes by, PANI towers are gained.

It is well known that wettability is a fundamental property of a solid surface and is influenced by the chemical composition and the topographical surface structure. Thus, surface wettability can be tuned by changing topographical surface structure^28^,^29^,^30^. It is reasonable to accept that the dosage of APTES which is the source of amino groups plays a decisive role in directing the PANI morphology. Small variation in APTES dosage results in significant changes in PANI morphology as shown above, which is expected to produce a pronounced effect on the final PANI surface wettability.

**Figure 7:**
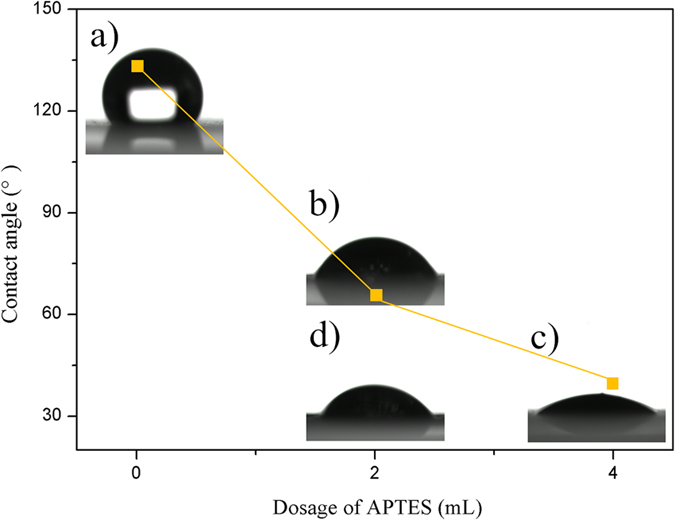
The dependence of dosage of APTES on the CA of (**a**) flowers, (**b**) tentacles, (**c**) nanofibers, and (**d**) towers.

**Figure 8:**
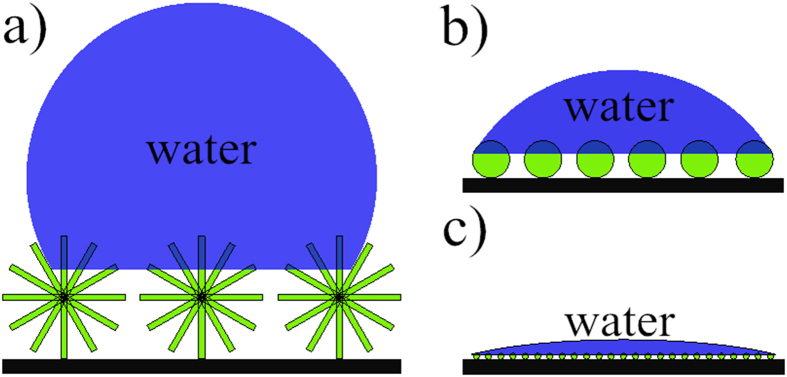
Possible mechanism for surface structure on the wetting behavior of PANI (**a**) flowers, (**b**) tentacles and towers, and (**c**) nanofibers.

Here, PANI surface wettability is evaluated by means of static water contact angle (CA) measurement. Figure 7 shows CA changes as a function of dosage of APTES and photographs of water droplet mounted on different PANI films. When the dosage of APTES is 0, 2, 4 mL, the corresponding CA is 133° ((a) flowers), 67~60° ((b) tentacles and (d) towers), and 37° (nanofibers). These CA measurements indicate that PANI can easily regulate surface wettability from hydrophobicity to hydrophilicity by simply altering its morphology. The notable change is well attributed to the synergistic effect of PANI dimension, hierarchy, and size. With increasing the dosage of APTES, the dimension of resulting PANI reduces from three to one. The dimensional reduction inevitably brings about the formation of simple hierarchical structures. Besides, PANI size varies from micron to nanometer level. For visual clarity, Fig. 8 depicts the possible mechanism of topographical surface structure on the wetting behavior. The air left in the nanostructures has been considered as the main factor to affect the surface wettability, which can effectively support the water to maintain droplet shape. The more air nanostructures have, the bigger CA they get^29^. There are more air existing among the PANI flowers (Fig. 8(a)) since PANI flowers have high dimension, complex hierarchy, and micron size, while the least air exists in the low dimensional, simple hierarchical, and nano-sized PANI nanofibers (Fig. 8(c)).

In summary, we have successfully realized PANI dimensional tuning through the addition of minute Fe_3_O_4_ microspheres decorated with different amounts of amino groups to aniline polymerization in 0.010 M HCl solution at room temperature, as well as with or without magnetic field. Novel PANI flowers, tentacles, nanofibers, and towers were respectively prepared. It was found that the quantity of amino groups on the surface of Fe_3_O_4_ microspheres determines the resulting PANI morphology. These PANI nanostructures exhibited obviously different surface wettability, which was assigned to the cooperative effect of their dimension, hierarchy, and size. Understanding PANI morphological tuning may enhance our knowledge of PANI nucleation and growth processes, and towards achieving more delicate morphological adjustment. This robust method described herein not only affords control of PANI dimensional regulation, but also paves the way to guide the other conducting polymers.

## Methods

### Synthesis of Fe_3_O_4_ microspheres

Fe_3_O_4_ microspheres were prepared through a solvothermal reaction. Briefly, FeCl_3_·6H_2_O (2.7 g) was dissolved in EG (80 mL) to form a clear solution, followed by the addition of NaAc (7.2 g) and PEG4000 (2.0 g). The mixture was stirred vigorously for 30 min and then sealed in a teflon-lined stainless-steel autoclave (100 mL). The autoclave was heated to and maintained at 200 °C for 8 h, then cooled to room temperature. The black Fe_3_O_4_ microspheres were washed several times with ethanol and deionized water, then dried at 60 °C for 12 h.

### Modification of Fe_3_O_4_ microspheres with amino groups

The amino-Fe_3_O_4_ microspheres were prepared by hydrolysis with APTES. Typically, Fe_3_O_4_ (100 mg) and APTES (2.0 or 4.0 mL) were dispersed in a mixed solution of ethanol (40 mL) and deionized water (40 mL). NH_3_·H_2_O (1.0 mL) as catalyst was added into the solution. Then, the solution was heated at reflux for 24 h at 70 °C to obtain (I) amino-Fe_3_O_4_ microspheres (2 mL APTES) and (II) amino-Fe_3_O_4_ microspheres (4 mL APTES).

### Synthesis of PANI bulks, flowers, tentacles, and nanofibers

In a typical run, Fe_3_O_4_ microspheres (10 mg, (I) amino-Fe_3_O_4_ microspheres: 10 mg, or (II) amino-Fe_3_O_4_ microspheres: 10 mg) and purified aniline (0.30 mL) were dispersed in a HCl solution (90 mL, 0.010 M) to form a homogeneous solution by using ultrasound for half an hour. Another HCl solution (10 mL, 0.010 M) containing APS (164.2 mg) was quickly injected into the above solution to oxidize aniline monomers. The polymerization was left to stand at 20 °C for 10 h. The resulting samples were washed with ethanol and deionized water several times, and PANI flowers, tentacles, and nanofibers were respectively obtained through magnetic separation.

An experiment was carried out and its reaction conditions were the same as those of above quintessential synthesis with the exception of in the absence of Fe_3_O_4_ microspheres. The resulting sediment was suction filtered with ethanol and deionized water until the suspension reached a neutral pH value. Eventually, PANI bulks were acquired.

### Synthesis of PANI towers

In a representative procedure, all procedures were the same as those of PANI tentacles except that a plane magnet (0.50 T) was placed on the side of the vessel with a distance of about 4 cm during the aniline polymerization.

### Characterization

Field emission scanning electron microscopy (FE-SEM) images were taken with a ZEISS MERLIN microscope. Samples dispersed in ethanol were deposited onto silicon wafers and sputtered with platinum by a JFC-1600 auto fine coater at a 20 mA current for 300 s prior to observation. Transmission electron microscopy (TEM) images were examined by a JEOL JEM-3010 microscope with Oxford 794-CCD camera at an accelerating voltage of 200 kV. Samples suspended in ethanol were dropped onto copper grids coated with a carbon support film before observation. The photographs of the water contact angle were recorded with an ultrapure water droplet of 8 μL on a JC2000D1 contact angle analyzer at room temperature. All the contact angle values were determined by using the Laplace-Young fitting mode.

## Additional Information

**How to cite this article**: Ma, Y. *et al.* Amino-Fe_3_O_4_ Microspheres Directed Synthesis of a Series of Polyaniline Hierarchical Nanostructures with Different Wettability. *Sci. Rep.* **6**, 33313; doi: 10.1038/srep33313 (2016).

## Supplementary Material

Supplementary Information
