# Supplementary material for: Amino-Fe3O4 Microspheres Directed Synthesis of a Series of Polyaniline Hierarchical Nanostructures with Different Wettability
Source: Sci Rep. 2016 Sep 16;6:33313. doi: 10.1038/srep33313 (PMC5025879; doi:10.1038/srep33313)
Supplement: Supplementary Information [file srep33313-s1.doc]

**Amino-Fe_3_O_4_ Microspheres Directed Synthesis of a Series of Polyaniline Hierarchical Nanostructures** **with Different Wettability**

Yong Ma, Yanhui Chen, Chunping Hou, Hao Zhang, Mingtao Qiao, Hepeng Zhang*, Qiuyu Zhang*

Key Laboratory of Applied Physics and Chemistry in Space of Ministry of Education, School of Science, Northwestern Polytechnical University, Xi'an 710072, P. R. China

**Materials**

Aniline (Hongyan Chemical Reagent Co., Ltd.) was purified by distilling it under reduced pressure and storing it in a refrigerator. Ferric chloride hexahydrate (FeCl_3_·6H_2_O; Hongyan Chemical Reagent Co., Ltd.), ethylene glycol (EG; Jinshan Chemical Reagent Co., Ltd.), sodium acetate (NaAc; Jinhua Chemical Reagent Co., Ltd.), polyethylene glycol 4000 (PEG4000; Kemiou Chemical Reagent Co., Ltd.), absolute ethanol (Fuyu Chemical Reagent Co., Ltd.), 3-aminopropyltriethoxysilane (APTES; Chenguang Chemical Reagent Co., Ltd.), ammonium hydroxide (NH_3_·H_2_O; Sanpu Chemical Reagent Co., Ltd.), and ammonium persulfate (APS; Hongyan Chemical Reagent Co., Ltd.) were of analytical grade and used as received. Deionized water was used throughout all the synthetic processes.


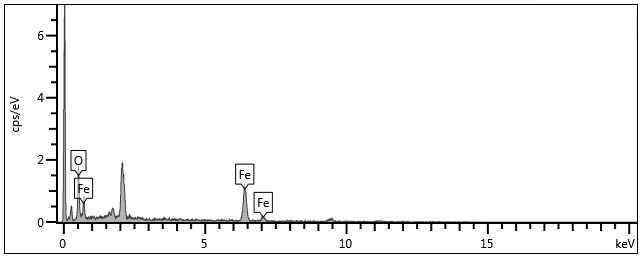


| element | wt% | wt% Sigma | atomic percent% |
| --- | --- | --- | --- |
| O | 16.73 | 0.91 | 41.23 |
| Fe | 83.27 | 0.91 | 58.77 |
| totals: | 100.00 |  | 100.00 |

Figure S1. EDS of Fe_3_O_4_ microspheres.


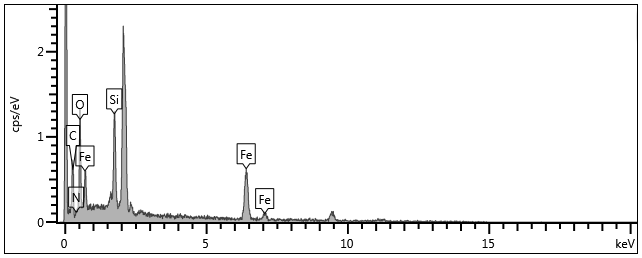


| element | wt% | wt% Sigma | atomic percent% |
| --- | --- | --- | --- |
| C | 25.34 | 1.51 | 46.07 |
| N | 1.80 | 1.50 | 2.81 |
| O | 19.00 | 0.95 | 25.94 |
| Si | 10.67 | 0.54 | 8.30 |
| Fe | 43.18 | 1.50 | 16.88 |
| totals: | 100.00 |  | 100.00 |

Figure S2. EDS of (I) amino-Fe_3_O_4_ microspheres.


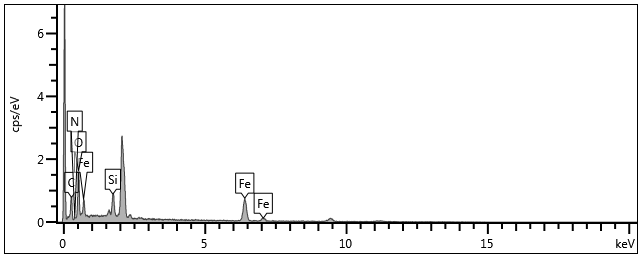


| element | wt% | wt% Sigma | atomic percent% |
| --- | --- | --- | --- |
| C | 22.83 | 0.91 | 42.11 |
| N | 2.74 | 0.87 | 4.34 |
| O | 21.77 | 0.63 | 30.14 |
| Si | 6.45 | 0.28 | 5.09 |
| Fe | 46.21 | 0.96 | 18.33 |
| totals: | 100.00 |  | 100.00 |

Figure S3. EDS of (II) amino-Fe_3_O_4_ microspheres.

**
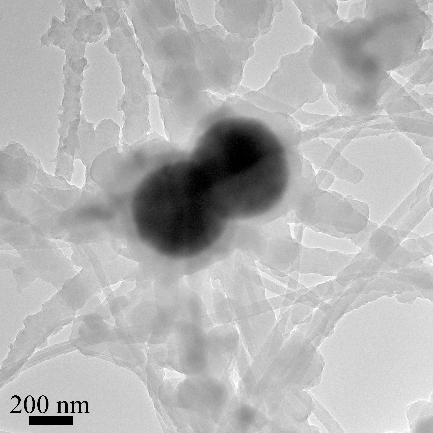
**

Figure S4. TEM images of PANI nanofibers obtained by adding (II) amino-Fe_3_O_4_ microspheres.

**
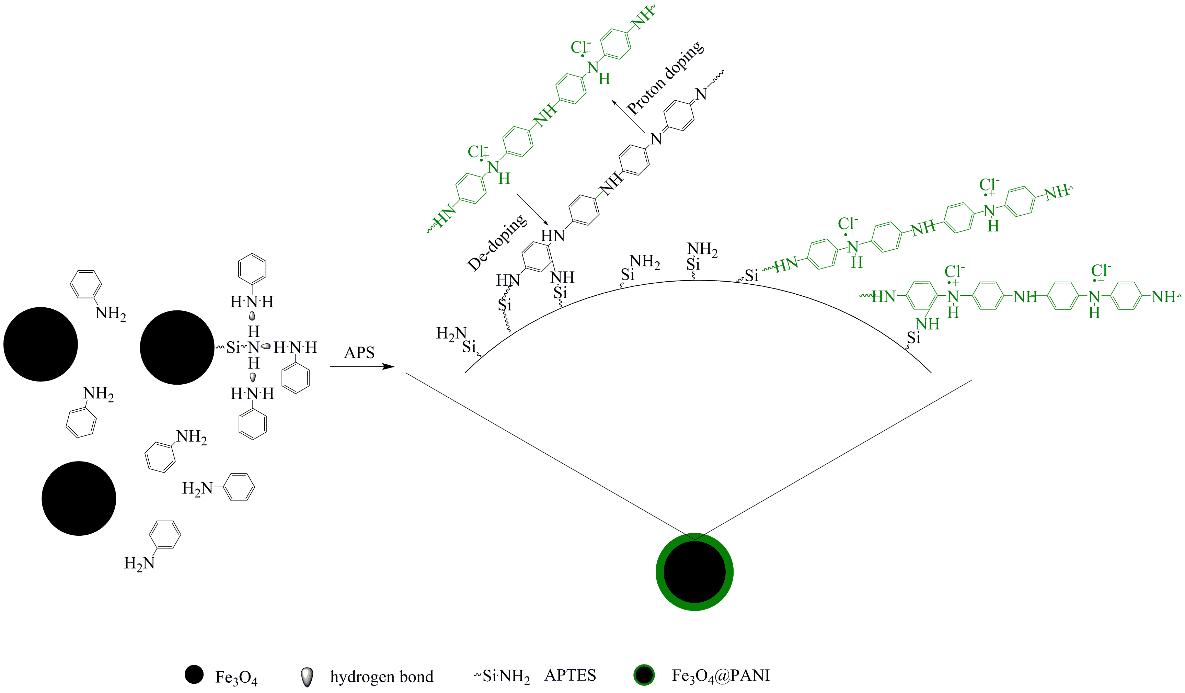
**

Figure S5. The illustration of possible hydrogen bonding effect between the amino-Fe3O4 microspheres and the amino groups of aniline and PANI.

**
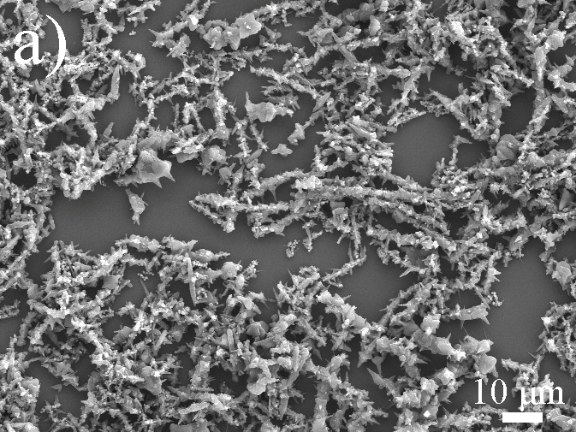

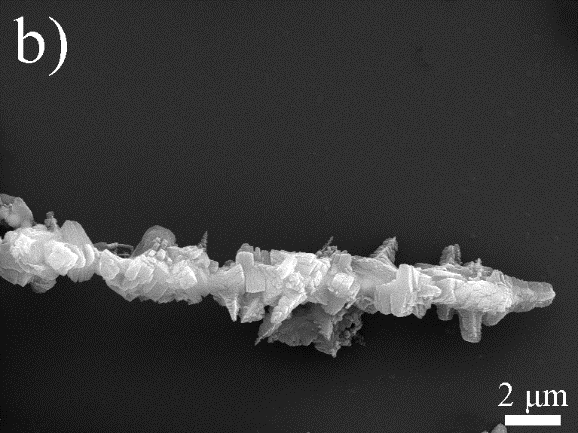

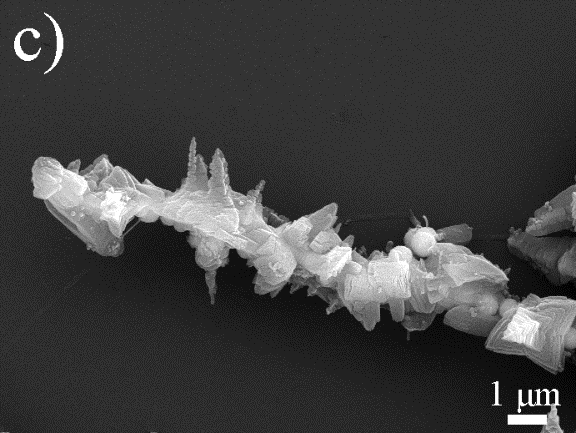
**

Figure S6. SEM image of PANI towers obtained by using (I) amino-Fe_3_O_4_ microspheres under an external magnetic field.
